# Icariin Alleviates Glucocorticoid-Induced Osteoporosis through EphB4/Ephrin-B2 Axis

**DOI:** 10.1155/2020/2982480

**Published:** 2020-05-17

**Authors:** Mi Huang, Ying Wang, Rui Peng

**Affiliations:** ^1^Hubei University of Chinese Medicine, Hubei, Wuhan 430000, China; ^2^South China Botanical Garden, Chinese Academy of Sciences, Key Laboratory of South China Agricultural Plant Molecular Analysis and Genetic Improvement, Key Laboratory of Guangdong Province Applied Botany, Guangzhou 510650, China; ^3^Gannan Normal University, Ganzhou, Jinagxi 341000, China

## Abstract

**Purpose:**

Glucocorticoid (GC) is the most important risk factor for osteoporosis (OP); in the present study, we examined the potential mechanism of icariin, a natural bioactive compound isolated from the traditional Chinese herbal *Epimedium*, for GC-induced OP to explore its potential therapeutic effect.

**Methods:**

We used a GC-induced OP mice model and treated with icariin. Pathological changes were measured by H&E staining, and the effects of icariin on osteoblasts and osteoclasts were measured by immunohistochemistry (IHC) staining and western blot (WB) analyses, while trabecular bone parameters were detected by micro-CT imaging *in vivo*.

**Results:**

The results showed that in GC-induced OP symptoms, icariin treatment significantly increased the density of the trabecular bone when exposed to GC, revealed by H&E staining and micro-CT imaging. IHC staining showed that GC-induced OP had a lower EphB4 expression and higher Ephrin-B2 expression, but icariin could promote EphB4 while suppressing Ephrin-B2 expression. The WB results also provided evidence of the same protein expression trend, showing that the osteoblast marker OCN and the EphB4 downstream factor RhoA in the GC group were decreased, while both OCN and RhoA expression were significantly increased and the Ephrin-B2 downstream factor Grb4 in in GC group was increased after icariin treatment.

**Conclusion:**

Icariin could improve the characteristics of OP through regulating the balance of the EphB4/Ephrin-B2 pathway. Further preclinical trial is needed to provide certainty of clinical benefits for OP patients.

## 1. Introduction

Osteoporosis (OP) is a skeletal disorder characterized by decreased bone strength and increased risk of fracture. OP can be divided into primary and secondary damage, and the secondary damage is mainly caused by any disease or drug that affects bone formation, among which glucocorticoid (GC) is the most important risk factor for OP. GC is widely used in the clinic due to its anti-inflammatory and immunosuppressive effects. However, long-term use of GC usually resulted in a 30%∼50% incidence of osteoporotic fractures, which seriously affects the quality of life of patients [1–5].

A number of signal pathways have been found participating in osteoblasts and osteoclasts in bone formation, and EphB4/Ephrin-B2 is the bidirectional signal axis that is separately expressed in the osteoblast and osteoclast cells [6], which produce positive or reverse signals. Ephrin-B2 inhibits osteoclast function through c-Fos/NFATc1, while EphB4 factor promotes osteoblast function by osteogenesis Dlx5, Osx, and Runx2 [7]. Similarly, Sema3A/Nrp, another signaling pathway between osteoblasts and osteoclasts, has been found to inhibit RANKL-induced osteoclast differentiation by inhibiting ITAM and RhoA signaling pathways [8]. Also, KEGG analysis found that EphB4 could participate in cytoskeleton development through the RhoA signaling pathway, while Ephrin-B2 could bind to Grb4 to generate inhibitory regulation. Nevertheless, the destruction of such signal axis causes functional changes of osteoblasts and osteoclasts, which may further lead to the formation barriers of osteocytes and the reduction of mineral deposits and increase the risk of formation of OP and fracture.

Recent studies have suggested that icariin could protect GC-induced OP [9], and icariin is found to participate in bone metabolism and synthesis through MAPK, RUN2, Rankl, and Wnt-catenin pathways and improve the differentiation of osteoblast precursor cells and osteoblast progression [10–15]. Interestingly, EphB4/Ephrin-B2 and Notch signaling pathways were revealed to play an important role in SRY-mediated male-specific artery formation during testosterone-induced XY gonad development [16]. However, icariin is a testosterone analogue [17], so it remains unclear whether the EphB4/Ephrin-B2 signaling axis is the key signaling mechanism for icariin. In the present study, we investigated the potential mechanism of icariin in GC-induced OP.

## 2. Materials and Methods

### 2.1. Animals

The C57/BL6 male mice (8 weeks, 21 ± 1.8 g) were used, supplied by the Experimental Animal Center of Tongji Medical College of Huazhong University of Science and Technology. The experiment complied the Regulations for the Administration of Affairs Concerning Experimental Animals by China (revised 2017). The study was approved by the Local Animal Ethics Committee of Wuhan First Hospital. The mice were randomly divided into 4 groups with 6 mice. Each group was treated for 60 days and followed by gavage for following four weeks according to the same protocol in [18]. The control group received continuous subcutaneous injection of normal saline at 5 mg/kg, followed by intragastric gavage with normal saline at 250 mg/kg/day; in the experimental group, 5 mg/kg prednisone sustained release agent was continuously injected subcutaneously, and the same amount of normal saline was gavage; in the drug treatment group, 5 mg/kg prednisone sustained release was injected subcutaneously continuously, and 250 mg/kg/day icariin (PS13012801, Purity: 99.50%, Chengdu PUSH Bio-Technology Co., Ltd.) was gavage. The positive control group was subcutaneously injected with the same amount of normal saline and gavage of icariin.

### 2.2. Histology Analysis

The right femurs and tibias were obtained and fixed at 4% formalin for at least 24 hrs and then immersed in 10% EDTA for 4 weeks, followed by embedding in paraffin. The tissues were cut to 4-*μ*m on slides. Following H&E staining, the sections were observed with an inverted microscope (Olympus, Tokyo, Japan).

### 2.3. Immunohistochemistry (IHC) Staining

The sections were deparaffinized in xylene followed by hydration in ethanol. Antigen retrieval was performed using citrate (pH 6.0) in a microwave. Then, the samples were blocked in bovine serum albumin for 1 hour. Primary antibodies EphB4 (bs-10659R) and Ephrin-B2 (bs-6046R), purchased from Beijing Bioss Biological Technology (Beijing, China), were incubated (1 : 200, 4°C, overnight), followed by incubation with a secondary antibody (HRP, DAKO, Carpinteria, CA, USA) for 1 hour. Diaminobenzidine (DAB-DAKO, Carpinteria, CA, USA) was used for positive visualization. The sections were observed with an inverted microscope (Olympus, Tokyo, Japan).

### 2.4. Western Blot (WB) Analysis

Whole proteins were isolated according to the kit protocol (KeyGen Biotech, Nanjing, China), and protein was then separated by 10% SDS-PAGE gels and transferred to polyvinyl difluoride (PVDF) membranes. Nonfat milk diluted in TBST was used for blocking for 1 hour at room temperature. Primary antibodies Grb4 (Proteintech, 10206-1-AP), RhoA (Proteintech, 10749-1-AP), and OCN (Proteintech, 23418-1-AP) (all from Wuhan SanYing, Wuhan) were incubated overnight at 4°C and then incubated with the anti-rabbit antibody for 2 hours at room temperature. The proteins were detected by the ECL detection kit, and relative expression levels were normalized to *β*-actin.

### 2.5. Micro-CT Imaging

After the mice in each group were sacrificed, the right tibia was quickly removed, and then the attached soft tissue was removed and the bone tissue was fixed in a 4% paraformaldehyde solution. The specimen was placed in a sample cup and fixed, and the trabecular microstructure of the tibia was scanned using an *in vitro* micro-CT tomography technique (SkyScan 1176, Institute of Hydrobiology, Chinese Academy of Sciences). The scanning conditions were set to a voltage of 70 kV, a scanning current of 200 *μ*A, a layer spacing of 14.80 *μ*m, a planar resolution of 300 ms, and a continuous scanning of about 808 layers. After the scan is completed, the sacral enlargement and the backbone area are selected as the region of interest (ROI). After the three-dimensional reconstruction threshold is set, the three-dimensional reconstruction of the ROI is performed. The airborne software CTan analysis is used. The main detection parameters are as follows: (1) bone mineral density parameters: BMD (bone mineral density), TMC (tissue mineral content), and BMC (bone mineral content); (2) bone structure parameters: BV/TV (bone volume/total volume), Tb.N (trabecular bone number), and Tb.Th (trabecular thickness) [19].

### 2.6. Statistical Analyses

One-way ANOVA followed by Tukey *post hoc* test was used for statistical analyses. The data were expressed as means ± SD. *P* values <0.05 were considered as statistically significant.

## 3. Results

### 3.1. Icariin Alleviates GC-Induced OP

**Figure 1:**
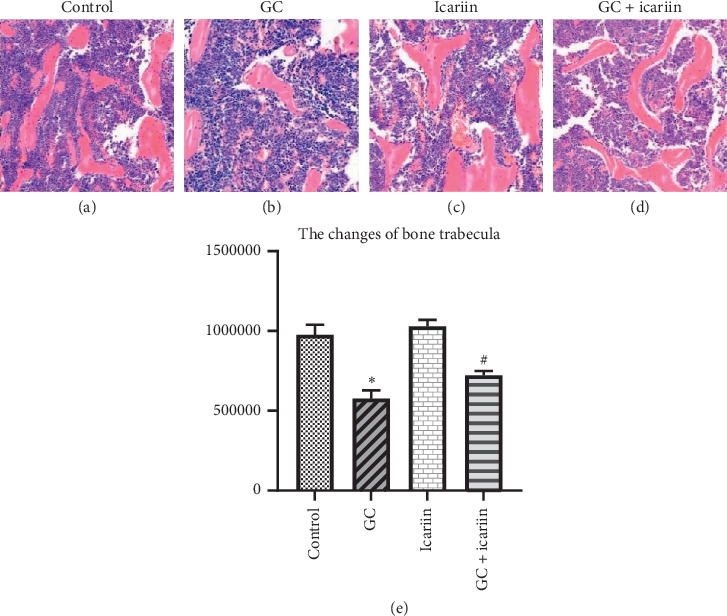
Icariin can alleviate the GC-induced OP. HE staining (200x) of control group (a), GC group (b), icariin group (c), and icariin treatment of GC-induced OP group (d). The GC treatment group showed a larger marrow and a decreasing number of trabecular bone; icariin treatment can reverse those changes (e). ^*∗*^*P* < 0.05 vs the control group and ^#^*P* < 0.05 vs the GC group.

HE staining (Figure 1) showed abundant, continuous, and dense trabecular bone in normal mice, but the GC treatment group showed a larger marrow cavity and a decreasing number of trabecular bones, revealing the typical characteristics of osteoporosis. The icariin treatment revealed reverses of those osteoporosis changes, suggesting icariin along having no changes as compared with the normal mice.

### 3.2. Icariin Improves Trabecular Bone Parameters in GC-Induced OP

**Figure 2:**
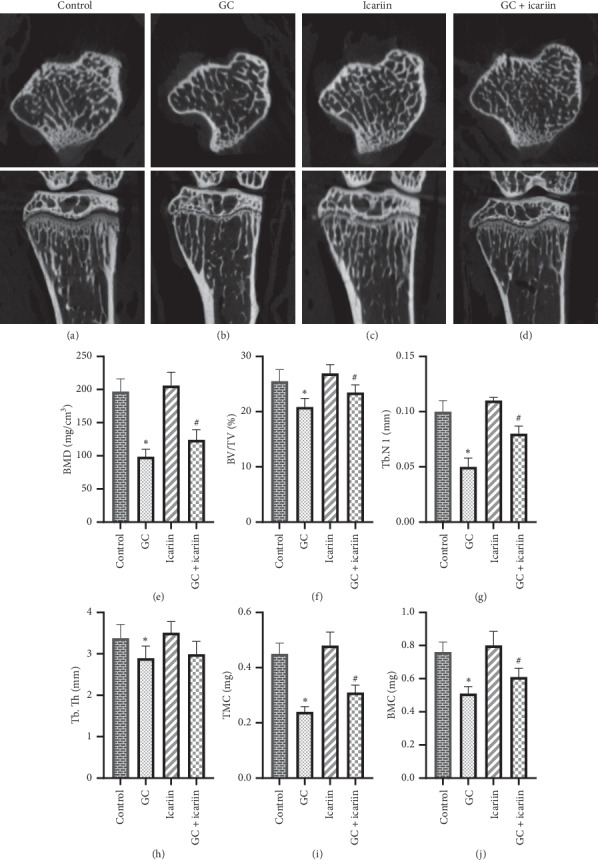
Icariin can improve trabecular bone parameters in GC-induced OP. Micro-CT was used to test trabecular bone parameters in the control group (a), GC group (b), icariin group (c), and icariin treatment of GC-induced OP group (d). Results on bone mineral density (BMD) (e), bone volume fraction (BV/TV) (f), trabecular number (Tb.N) (g), trabecular thickness (Tb.Th) (h), tissue mineral content (TMC) (i), and trabecular bone mineral content (Tb.BMC) (j). The GC treatment made significant negative effects on microstructural quantitative parameters, while icariin can prevent those changes. ^*∗*^*P* < 0.05 vs the control group and ^#^*P* < 0.05 vs the GC group.

Trabecular bone parameters were analyzed by using micro-CT (Figure 2), and the results showed that GC treatment had significantly negative effects on BV/TV, Tb.N, Tb.Th, BMD, TMC, and BMC, while icariin treatment significantly reduced the negative effects of structural properties of the trabecular bone on BV/TV, Tb.N, BMD, TMC, and BMC. Icariin along had no structural changes as compared with the normal mice.

### 3.3. Icariin Elevates EphB4 While Suppressing Ephrin-B2 Expression in GC-Induced OP

**Figure 3:**
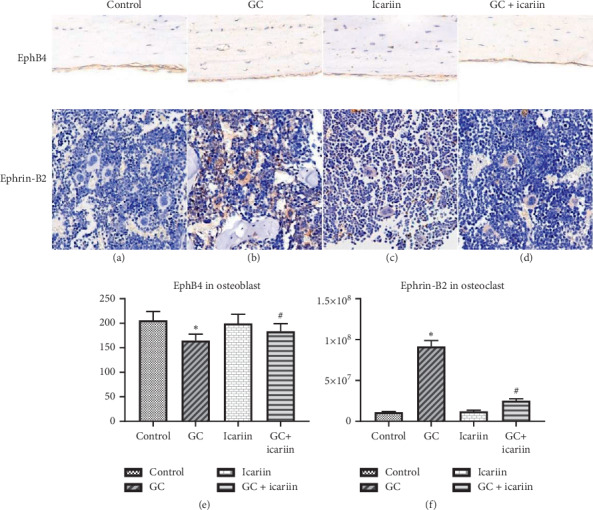
Icariin can promote EphB4 while suppress Ephrin-B2 expression in GC-induced OP. IHC staining of Ephrin-B2 and EphB4 in the control group (a), GC group (b), icariin group (c), and icariin treatment of GC-induced OP group (d). The WB results showed that the GC treatment decreased EphB4 expression and increased Ephrin-B2 expression compared with the control group, and the GC + icariin group showed the opposite result compared with the GC group (e and f). ^*∗*^*P* < 0.05 vs the control group and ^#^*P* < 0.05 vs the GC group.

To further explore the relationship between EphB4 and Ephrin-B2 in GC-induced OP, IHC staining (Figure 3) was used, revealing GC-induced Ephrin-B2 expression, while after icariin treatment, Ephrin-B2 expression was significantly decreased. We further found that GC can suppress EphB4 expression, while after icariin treatment, Ephrin-B2 expression was significantly increased. The WB results also confirmed the protein expression trend.

### 3.4. Icariin Alleviates GC-Induced OP through the EphB4/Ephrin-B2 Axis

**Figure 4:**
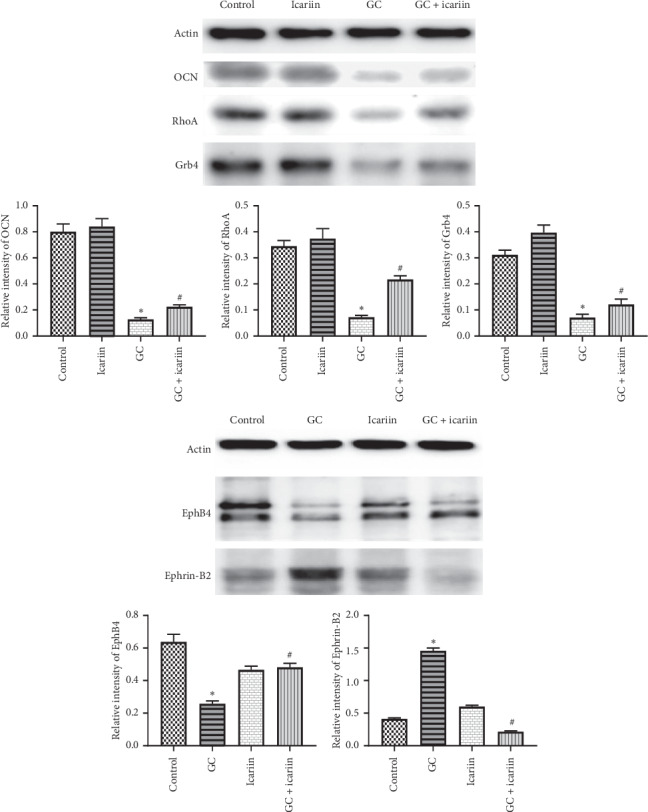
Icariin alleviates GC-induced OP through the EphB4/Ephrin-B2 axis. WB analysis of the osteoblast marker OCN, EphB4 downstream factor RhoA, and Ephrin-B2 downstream factor Grb4. The result showed that the osteoblast marker OCN, the EphB4 downstream factor RhoA, and the Ephrin-B2 downstream factor Grb4 in the GC group were decreased, while after icariin treatment, their expressions were all significantly increased. Protein expression levels were normalized to *β*-actin. ^*∗*^*P* < 0.05 vs the control group and ^#^*P* < 0.05 vs the GC group.

We also examined the signaling pathway involved in GC-induced OP. The WB results (Figure 4) showed that the osteoblast marker OCN and the EphB4 downstream factor RhoA in the GC group were decreased, while after icariin treatment, both OCN and RhoA expression were significantly increased. Furthermore, we found that the Ephrin-B2 downstream factor Grb4 in the GC group was decreased, while after icariin treatment, Grb4 expression was significantly increased. The results suggested that icariin might regulate the balance of the EphB4/Ephrin-B2 axis, thus promoting the recovery of GC-induced OP.

## 4. Discussion

The clinical treatment for GC-induced OP relies on drugs including calcium supplementation, vitamin D, bisphosphonates, hormones, calcitonin, and fluoride. However, the effect of drug therapy is still unsatisfactory [20]. Icariin, a natural product from traditional Chinese medicine (TCM), has been studied for treatment of osteoporosis [21], and its pharmacological function remains unclear. In the present study, we found that icariin could alleviate GC-induced OP through regulating the balance of the EphB4/Ephrin-B2 axis, thus enhancing the recovery of OP.

GC is an important pathogenetic factor for OP and affects the differentiation and longevity of osteoclasts and osteoblasts through redox pathways [22]. GC reduces replication of osteoblastic lineage cells and inhibits osteogenic differentiation or mineralization, resulting in the osteoblasts in an immature state, thereby reducing their numbers and function. GC can also indirectly inhibit osteogenic differentiation and promote adipogenic differentiation in stromal cells [23]. Moreover, GC also increases the function and number of osteoclasts and their survival time, such as caspase-3 can prolong its life and M-CSF can enhance its activity [24, 25].

The Eph receptor is a primary component of the tyrosine kinase receptor family. EphB4/Ephrin-B2 signaling is an important axis which regulates the functional balance of osteoblasts and osteoclasts [26]. The regulation was via the transmembrane ligand, such as Ephrin-2 on osteoclasts and receptor tyrosine kinase and EphB4 on osteoblasts [7]. Previous studies suggested that EphB4 receptor activation by Ephrin-B2 could affect resorption factors and their activities in the subchondral bone [27]. Ephrin-B2 treatment of osteoarthritic chondrocytes increases the gene expression levels of type II collagen in these cells [28], suggesting that Ephrin-B2 also plays a role in regulating chondrocyte differentiation. It was also reported that osteoclastogenesis could be attenuated through bidirectional EphB4/Ephrin-B2 signaling *in vitro* [29], while another study found that Claudin 11 may regulate bone homeostasis via bidirectional EphB4/Ephrin-B2 signaling [30]. In the present study, we found that GC treatment could induce typical characteristics of osteoporosis, while icariin treatment reversed those osteoporosis changes and demonstrated a therapeutic effect of icariin on GC-induced OP. Further study found that both EphB4 and Ephrin-B2 participated in the pathological changes of OP, which is consistent with previous studies that EphB4/Ephrin-B2 signaling also promotes the differentiation of osteoblasts and osteoclasts [26], indicating an imbalance expression of EphB4 and Ephrin-B2 could be a potential mechanism which might disturb the function of osteoblasts and osteoclasts.

Moreover, through KEGG analysis, we found that EphB4 could act on the RhoA signaling pathway; i.e., Ephrin-B2 could bind to Grb4 and might further regulate cytoskeleton development. To test whether RhoA and Grb4 pathways are involved in the EphB4/Ephrin-B2 signaling, we detected their protein levels and found lower expression of RhoA but higher Grb4 levels in GC-induced OP, while treatment with icariin reversed the expression, which implied that EphB4 could further inhibit osteoblasts through the RhoA signaling pathway, while Ephrin-B2 regulated Grb4 to promote osteoclast function. As their functional properties are similar to testosterone [17], our findings provided new evidence that icariin regulates the expression of EphB4/Ephrin-B2 and maintains the functional balance of osteoblasts and osteoclasts through RhoA and Grb4, respectively, to protect the bone structure.

## 5. Conclusions

In the present study, we found icariin could improve characteristics of OP through regulating the balance of EphB4/Ephrin-B2 to protect the bone structure. A further preclinical trail might be needed to provide certainty of clinical benefits for OP patients.

## Data Availability

The data used to support the findings of this study are included within the article. Any further data can be made available from the corresponding author upon request.

## Abbreviations

OP: Osteoporosis
GC: Glucocorticoid
IHC: Immunohistochemistry
WB: Western blot
ROI: Region of interest
BMD: Bone mineral density
TMC: Tissue mineral content
BMC: Bone mineral content
BV/TV: Bone volume/total volume
Tb.N: Trabecular bone number
Tb.Th: Trabecular thickness.
